# *Marchantia polymorpha*, a New Model Plant for Autophagy Studies

**DOI:** 10.3389/fpls.2019.00935

**Published:** 2019-07-17

**Authors:** Takuya Norizuki, Takehiko Kanazawa, Naoki Minamino, Hirokazu Tsukaya, Takashi Ueda

**Affiliations:** ^1^Department of Biological Sciences, Graduate School of Science, The University of Tokyo, Tokyo, Japan; ^2^Division of Cellular Dynamics, National Institute for Basic Biology, Okazaki, Japan; ^3^Department of Basic Biology, The Graduate University for Advanced Studies (SOKENDAI), Okazaki, Japan

**Keywords:** autophagy, ATG, plant evolution, gene duplication, *Marchantia polymorpha*

## Abstract

Autophagy is a catabolic process for bulk and selective degradation of cytoplasmic components in the vacuole/lysosome. In *Saccharomyces cerevisiae, ATG* genes were identified as essential genes for autophagy, and most *ATG* genes are highly conserved among eukaryotes, including plants. Although reverse genetic analyses have revealed that autophagy is involved in responses to abiotic and biotic stresses in land plants, our knowledge of its molecular mechanism remains limited. This limitation is partly because of the multiplication of some *ATG* genes, including *ATG8*, in widely used model plants such as *Arabidopsis thaliana*, which adds complexity to functional studies. Furthermore, due to limited information on the composition and functions of the *ATG* genes in basal land plants and charophytes, it remains unclear whether multiplication of *ATG* genes is associated with neofunctionalization of these genes. To gain insight into the diversification of *ATG* genes during plant evolution, we compared the composition of *ATG* genes in plants with a special focus on a liverwort and two charophytes, which have not previously been analyzed. Our results showed that the liverwort *Marchantia polymorpha* and the charophytes *Klebsormidium nitens* and *Chara braunii* harbor fundamental sets of *ATG* genes with low redundancy compared with those of *A. thaliana* and the moss *Physcomitrella patens*, suggesting that multiplication of *ATG* genes occurred during land plant evolution. We also attempted to establish an experimental system for analyzing autophagy in *M. polymorpha*. We generated transgenic plants expressing fluorescently tagged MpATG8 to observe its dynamics in *M. polymorpha* and produced autophagy-defective mutants by genome editing using the CRISPR/Cas9 system. These tools allowed us to demonstrate that MpATG8 is transported into the vacuole in an MpATG2-, MpATG5-, and MpATG7-dependent manner, suggesting that fluorescently tagged MpATG8 can be used as an autophagosome marker in *M. polymorpha*. *M. polymorpha* can provide a powerful system for studying the mechanisms and evolution of autophagy in plants.

## Introduction

Autophagy is a highly conserved catabolic process in eukaryotes for degrading and recycling cytoplasmic components. Among several types of autophagy reported thus far, the molecular mechanisms and physiological significance of macroautophagy have been the most intensively studied (Mizushima et al., 2011). During macroautophagy (hereafter simply referred to as “autophagy”), a cup-shaped membrane sac called the isolation membrane or phagophore elongates and sequesters cytoplasmic components, and its edge is closed to form a double-membrane-bounded structure called the autophagosome (Baba et al., 1994). The outer membrane of the autophagosome fuses with the vacuolar/lysosomal membrane, and a single-membraned structure called the autophagic body is released into the luminal space of the vacuole/lysosome to be degraded by lytic enzymes (Takeshige et al., 1992; Baba et al., 1994, 1995). In addition to bulk degradation, certain substrates, including organelles, are selectively recognized and degraded by autophagy (Johansen and Lamark, 2011). In *Saccharomyces cerevisiae*, autophagy-related processes are also involved in biosynthetic delivery; the newly synthesized precursor form of aminopeptidase I is transported into the vacuole by small double-membrane-bounded vesicles (Baba et al., 1997), which are formed through a mechanism similar to autophagy (Harding et al., 1996; Scott et al., 1996).

In the 1990s, several groups identified *APG*/*AUT*/*CVT* genes (later unified under the name *ATG*) as essential genes for autophagy or autophagy-related processes in *S. cerevisiae* (Tsukada and Ohsumi, 1993; Thumm et al., 1994; Harding, 1995; Klionsky et al., 2003). Atg proteins are initially recruited to and function at the preautophagosomal structures or phagophore assembly site (PAS) in a hierarchical manner (Suzuki et al., 2001, 2007). Based on their functions, the essential genes for autophagosome formation in *S. cerevisiae* are classified into four groups: (1) the Atg1 complex, (2) phosphatidylinositol 3-kinase (PI3K) complex, (3) Atg9 cycling system, and (4) ubiquitin-like conjugation systems. The Atg1 complex, consisting of Atg1, Atg13, Atg17, Atg29, and Atg31, is one of the most upstream Atg factors and is recruited to the PAS under the regulation of various cellular signals (Noda and Ohsumi, 1998; Suzuki et al., 2007; Kamada et al., 2010). The Atg1 complex phosphorylates various substrates, including Atg9, and acts as a scaffold for downstream Atg proteins (Suzuki et al., 2007, 2015; Papinski et al., 2014; Harada et al., 2019). The PI3K complex comprises Atg6, Atg14, Vps15, and Vps34. The PI3K complex generates phosphatidylinositol 3-phosphate (PI3P) at the PAS, which leads to recruitment of PI3P-binding proteins such as the Atg2–Atg18 complex (Kihara et al., 2001; Obara et al., 2008). The Atg9 cycling system involves Atg2, Atg9, and Atg18. Atg9 is a multimembrane-spanning protein that shuttles between the PAS and mobile structures derived from the Golgi and provides a membrane source during the early step of autophagosome formation (Mari et al., 2010; Yamamoto et al., 2012). The Atg2–Atg18 complex is recruited to the edge of the isolation membrane with Atg9 and probably regulates recycling of Atg9 from the PAS (Reggiori et al., 2004; Graef et al., 2013; Suzuki et al., 2013). Atg2 was also recently shown to have membrane tethering and lipid transfer activities, which are crucial for expansion of the isolation membrane (Chowdhury et al., 2018; Gomez-Sanchez et al., 2018; Kotani et al., 2018; Osawa et al., 2019). Furthermore, mammalian WIPI2b, which is homologous to Atg18, interacts with and recruits ATG16L1a (homologous to Atg16) to regulate the site of lipidation of LC3 (homologous to Atg8) (Fujita et al., 2008b; Dooley et al., 2014). Two ubiquitin-like conjugation systems involve Atg3, Atg4, Atg5, Atg7, Atg8, Atg10, Atg12, and Atg16. Atg12 is conjugated to Atg5 by E1-like Atg7 and E2-like Atg10, and Atg12–Atg5 forms a complex with Atg16 (Mizushima et al., 1998, 1999; Shintani et al., 1999; Tanida et al., 1999; Kuma et al., 2002). Atg8 is cleaved by Atg4, and glycine is exposed at its C-terminus (Kirisako et al., 2000). This glycine is conjugated with phosphatidylethanolamine through the actions of E1-like Atg7, E2-like Atg3, and the E3-like Atg12–Atg5–Atg16 complex (Ichimura et al., 2000; Hanada et al., 2007). Lipidated Atg8 is recruited to the autophagosomal membrane, which functions in expansion and closure of the isolation membrane (Abeliovich et al., 2000; Nakatogawa et al., 2007; Fujita et al., 2008a; Xie et al., 2008; Tsuboyama et al., 2016). Atg8 also interacts with cargo receptors/adaptors via Atg8-interacting motifs (AIMs) or LC3-interacting regions (LIRs), which mediate the effective degradation of cargos (Noda et al., 2010; Nguyen et al., 2016; Padman et al., 2019). A recent study also demonstrated that ubiquitin-interacting motif (UIM)-like sequences are also recognized by Atg8 (Marshall et al., 2019).

Most *ATG* genes are also conserved in plants, and deletion of many *ATG* genes in *Arabidopsis thaliana* results in defective autophagy, suggesting that ATG proteins in *A. thaliana* have the same functions as those in *S. cerevisiae* and mammals (Doelling et al., 2002; Hanaoka et al., 2002; Yoshimoto et al., 2004; Thompson et al., 2005; Xiong et al., 2005; Inoue et al., 2006; Phillips et al., 2008; Chung et al., 2010; Suttangkakul et al., 2011; Li et al., 2014; Young et al., 2019). Genetic analyses have revealed that autophagy is involved in responses to abiotic and biotic stresses (Doelling et al., 2002; Liu et al., 2005, 2009; Xiong et al., 2007; Zhou et al., 2013; Chen et al., 2015). Whereas almost all *atg* mutants of *A. thaliana* are fertile under favorable growth conditions, autophagy is required for male gamete differentiation in *Oryza sativa* and *Physcomitrella patens* (Kurusu et al., 2014; Sanchez-Vera et al., 2017). Furthermore, selective degradation of various organelles, including peroxisomes, by autophagy has been reported (Kim et al., 2013; Shibata et al., 2013; Yoshimoto et al., 2014); the molecular mechanisms of this phenomenon, however, remain largely unknown (Yoshimoto and Ohsumi, 2018). The difficulty in studying autophagy in plants is partly due to the genetic redundancy of key *ATG* genes in model plants, including *A. thaliana*. For example, *A. thaliana, O. sativa*, and *P. patens* harbor nine, four, and six *ATG8* homologs, respectively, which makes it difficult to unravel the functions of ATG8 proteins in these plants (Kellner et al., 2017). Although functional differentiation among mammalian *ATG8* homologs has been reported (Weidberg et al., 2010), it remains completely unknown whether each *ATG8* homolog in plants acts at a different step in autophagy. The significance of the duplication of *ATG* genes during plant evolution also remains obscure, since information from basal land plants and algal species remains sparse. To obtain more insights into the diversification and evolution of autophagy in the plant lineage, information from charophytes, the closest living relatives of land plants, and from additional bryophytes would be needed.

In this study, we identified homologs of *ATG* genes in the basal land plants *Marchantia polymorpha* and *P. patens* and the charophytes *Klebsormidium nitens* and *Chara braunii*. A comparison among these species, as well as *Chlamydomonas reinhardtii* and *A. thaliana*, indicated that *M. polymorpha* shares a common set of *ATG* genes with low redundancy compared with those of other land plants; this finding suggested that *M. polymorpha* would be a good system to investigate the molecular mechanisms and physiological significance of autophagy in land plants. Many molecular genetic techniques and cell biological tools have been established for *M. polymorpha* (Era et al., 2009; Ishizaki et al., 2016; Kanazawa et al., 2016; Minamino et al., 2018), which would also be a good reason to use this plant for autophagy studies. In this study, as the first step toward the study of autophagy in *M. polymorpha*, we generated transgenic plants expressing fluorescently tagged MpATG8 proteins to monitor autophagosomes and produced the autophagy-defective mutants Mp*atg2*, Mp*atg5*, and Mp*atg7* to analyze the effect of defective autophagy on thallus development and their responses to nutrient starvation. Our results indicated that the number of *ATG* genes gradually increased during plant evolution. We also succeeded in monitoring the dynamics of MpATG8, which was transported into the vacuole in an MpATG2-, MpATG5-, and MpATG7-dependent manner. These tools would be useful for future studies to understand the basic mechanisms and physiological significance of autophagy in plants.

## Results

### Identification of Orthologs of Core Autophagy Machinery Components in Plants

Extensive studies using *S. cerevisiae* have revealed that *ATG1*–*10, 12*–*14, 16*–*18, 29*, and *31*, and *VPS15* and *VPS34* are required for autophagosome formation (Mizushima et al., 2011). Although some *ATG* genes are highly duplicated in angiosperms, the precise significance of the expansion of *ATG* genes remains obscure. To gain insight into this phenomenon, we searched the genome sequences of charophytes (*K. nitens* and *C. braunii*) and bryophytes (*M. polymorpha* and *P. patens*) for homologs of *ATG* genes, a number of which were then compared with those of *A. thaliana* and *C. reinhardtii*. As a query, we used the sequences of the *ATG* genes of *A. thaliana* (Shemi et al., 2015; Liu et al., 2018). We also examined *ATG11* and *ATG101*. *ATG11* is not required for starvation-induced bulk autophagy but is essential for selective autophagy in *S. cerevisiae* (Kim et al., 2001); Atg11 interacts with Atg1 and cargo receptors, which is crucial for selective autophagy (Yorimitsu and Klionsky, 2005; Farre et al., 2008; Okamoto et al., 2009). Atg101 forms a complex with ULK1 (homologous to Atg1) in mammalian cells, although *S. cerevisiae* does not harbor a homolog of *ATG101* (Hosokawa et al., 2014; Mercer et al., 2014). In *A. thaliana*, ATG11 and ATG101 are thought to form a complex with ATG1, and the *atg11* mutant exhibits similar phenotypes to those of other *atg* mutants (Li et al., 2014), suggesting that ATG11 and ATG101 are involved in general autophagy in plants. Therefore, *ATG11* and *ATG101* were also included among the “core autophagy machinery genes” in this study.

As shown in Table 1, almost all core autophagy machinery genes were highly conserved among the plants we investigated, although *ATG16* homologs in *C. reinhardtii* (Shemi et al., 2015) and *ATG2* and *ATG10* homologs in *C. braunii* were not detected. Homologs of *ATG17, ATG29*, and *ATG31* were not detected in this study, consistent with previous studies (Kawamata et al., 2005; Li et al., 2014). While a considerable number of *ATG* genes are present in *A. thaliana* and *P. patens, C. reinhardtii*, charophytes, and *M. polymorpha* possess only one gene for each core autophagy machinery component, with a few exceptions; multiple genes for *ATG8* and *ATG18* exist in the genomes of these plants with lower redundancy than *A. thaliana* and *P. patens* (Table 1). Thus, core autophagy machinery genes have seemingly expanded gradually during plant evolution.

**Table 1:** Composition of core autophagy machinery genes in Viridiplantae.

		ATG1 complex				PI3K complex				ATG9 cycling system			Ubiquitin-like conjugation system							
Species	Class	*ATG1*	*ATG11*	*ATG13*	*ATG10h*	*ATG6*	*ATG14*	*VPS15*	*VPS34*	*ATG2*	*ATG9*	*ATG18*	*ATG3*	*ATG4*	*ATG5*	*ATG7*	*ATG8*	*ATG10*	*ATG12*	*ATG16*
*C. reinhardtii*	Chlorophyceae	1	1^1^	1	1^2^	1	1^2^	1	1	1	1	2	1	1	1	1	1	1	1	ND
*K. nitens*	Klebsormidiophyceae	1	1	1	1	1	1	1	1	1	1	3	1	1	1	1	1	1	1	1
*C. braunii*	Charophyceae	1	2	1	1	1	1	1	1	ND	1	2	1	1	1	2	2	ND	1	1
*M. polymorpha*	Marchantiopsida	1	1	1	1	1	1	1	1	1	1	4	1	1	1	1	2	1	1	1
*P. patens*	Bryopsida	3	2	2	1	2	1	2	1	1	2	8	1	2	1	1	6^3^	1	1	1
*A. thaliana*	Magnoliopsida	4^4^	1^5^	2	1^5^	1	2^6^	1	1	1	1	8	1	2	1	1	9	1	2	1

*Numbers of core autophagy machinery genes are shown. “ND,” not detected in our analysis. For *A. thaliana* and *C. reinhardtii* homologs, we referred to the previous study (Shemi et al., 2015). 1, Identified in this study; 2, Jiang et al., 2012; 3, Kellner et al., 2017; 4, Suttangkakul et al., 2011; 5, Li et al., 2014; 6, Liu et al., 2018.*

Recently, Pang et al. (2019) reported that *ATG10* has been lost in quite a few lineages of eukaryotes, including Pichiaceae and the SAR supergroup, which comprises stramenopiles, alveolates, and Rhizaria. As summarized in Table 1, we detected an *ATG10* homolog in *K. nitens* but not in *C. braunii*. We also looked for *ATG10* homologs in other charophytes whose genome and/or transcriptome information was available (*Spirogyra pratensis, Nitella mirabilis, Coleochaete orbicularis*, and *Mesostigma viride*) and found that these algae, except for *N. mirabilis*, possess *ATG10* homologs. This distribution of *ATG10* suggested that secondary loss of *ATG10* occurred in Charophyceae (Table 2). The ATG10 protein is an E2-like enzyme required for covalent linkage between the C-terminal glycine residues of ATG12 and ATG5 (Mizushima et al., 1998; Shintani et al., 1999; Suzuki et al., 2005; Phillips et al., 2008; Chung et al., 2010). In *Toxoplasma gondii* and *Komagataella phaffii*, which do not possess *ATG10* homologs, ATG12 forms a noncovalent complex with ATG5, which does not require the C-terminal glycine of ATG12 (Pang et al., 2019). Intriguingly, the ATG12 of *C. braunii* does not harbor glycine at the C-terminus, although the glycine residue is conserved at the C-terminus of *N. mirabilis* ATG12 (Table 2). These features could reflect a similar mechanism of complex formation between ATG12 and ATG5 in *C. braunii*; ATG12 might form a noncovalent complex with ATG5.

**Table 2:** *ATG10* and *ATG12* homologs in charophytes.

Species	Class	ATG10	C-terminal Gly in ATG12
*M. viride*	Mesostigmatophyceae	1	+
*K. nitens*	Klebsormidiophyceae	1	+
*C. braunii*	Charophyceae	ND	–
*N. mirabilis*	Charophyceae	ND	+
*C. orbicularis*	Coleochaetophyceae	1	+
*S. pratensis*	Zygnematophyceae	1	+

*“ND,” not detected in our analysis; +, conserved; -, not conserved.*

**FIGURE 1:**
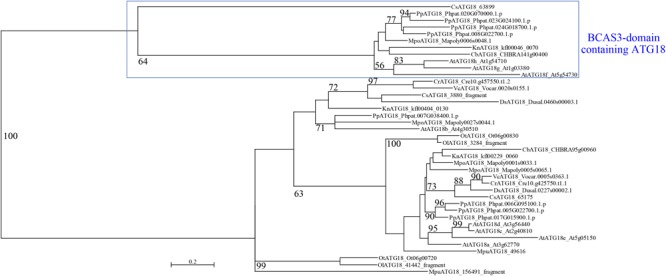
Phylogenetic analysis of ATG18 in Viridiplantae. The maximum-likelihood phylogenetic analysis was performed using amino acid sequences of ATG18 in Viridiplantae. Bootstrap probability with at least 50% support is indicated as percentage on each branch. At, *A. thaliana*; Pp, *P. patens*; Mpo, *M. polymorpha*; Cb, *C. braunii*; Kn, *K. nitens*; Cr, *C. reinhardtii*; Ds, *D. salina*; Vc, *V. carteri*; Cs, *C. subellipsoidea*; Mpu, *M. pusilla*; Ol, *O. lucimarinus*; Ot, *O. tauri*.

*A. thaliana* and *Nicotiana tabacum* are reported to possess two types of ATG18: conventional ATG18, which is similar to yeast and mammalian ATG18, and plant-unique ATG18, which harbors the BCAS3 domain at the C-terminus (Xiong et al., 2005; Zhou et al., 2015). All of the land plant species we analyzed in this study harbor both types of ATG18, whereas *C. reinhardtii* does not possess plant-unique ATG18 (Table 3). We then investigated whether plant-unique ATG18 is found in other chlorophytes. Although we did not detect ATG18 with the BCAS3 domain in *Dunaliella salina, Volvox carteri, Micromonas pusilla, Ostreococcus lucimarinus*, and *Ostreococcus tauri, Coccomyxa subellipsoidea* harbored this type of ATG18. Canonical *ATG18* homologs were found in all of these species (Table 3). Therefore, ATG18 containing the BCAS3 domain seems to have been acquired before the emergence of Streptophyta, which was also supported by the result of phylogenetic analysis (Figure 1).

**Table 3:** *ATG18* homologs in Viridiplantae.

		ATG18	
Species	Class	BCAS3-domain lacking	BCAS3-domain containing
*C. reinhardtii*	Chlorophyceae	2	ND
*K. nitens*	Klebsormidiophyceae	2	1
*C. braunii*	Charophyceae	1	1
*M. polymorpha*	Marchantiopsida	3	1
*P. patens*	Bryopsida	4	4
*A. thaliana*	Magnoliopsida	5	3
*D. salina*	Chlorophyceae	2	ND
*V. carteri*	Chlorophyceae	2	ND
*C. subellipsoidea*	Trebouxiophyceae	2	1
*M. pusilla*	Mamiellophyceae	2	ND
*O. lucimarinus*	Mamiellophyceae	2	ND
*O. tauri*	Mamiellophyceae	2	ND

*“ND,” not detected in our analysis.*

### Dynamics of MpATG8 in *M. polymorpha*

**FIGURE 2:**
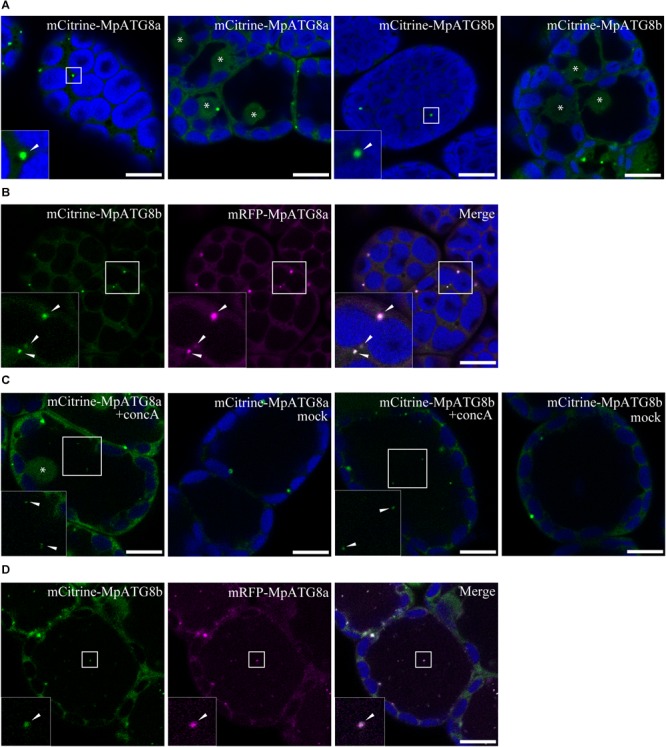
Subcellular localization of MpATG8 members. **(A)** MpATG8a and MpATG8b were localized to punctate structures in the cytosol, indicated by arrowheads. Fluorescence was also detected in the nucleus, indicated by asterisks. **(B)** mRFP-MpATG8a and mCitrine-MpATG8b colocalized to the punctate structures in the cytosol. Arrowheads indicate punctate compartments with mRFP-MpATG8a and mCitrine-MpATG8b. **(C)** MpATG8-positive puncta observed in the vacuoles upon concA treatment. The asterisk and arrowheads indicate the nucleus and puncta in the vacuole, respectively. **(D)** Colocalization of mRFP-MpATG8a and mCitrine-MpATG8b at punctate structures in the vacuole upon concA treatment. Arrowheads indicate punctate compartments with mRFP-MpATG8a and mCitrine-MpATG8b in the vacuole. The insets are magnified images of the boxed regions. Green, magenta, and blue show fluorescence from mCitrine, mRFP, and autofluorescence of chlorophyll, respectively. Scale bars = 10 μm.

Because of the low genetic redundancy of ATG components (Table 1) and molecular genetic tools available (Ishizaki et al., 2016), *M. polymorpha* is expected to be a good model for understanding the fundamental molecular mechanisms of autophagy in land plants. As the first step of the autophagy study using *M. polymorpha*, we generated transgenic plants expressing fluorescently tagged MpATG8 proteins under the regulation of their own promoters. ATG8 localizes to the isolation membrane during autophagosome formation and stays on the inner membranes of autophagosomes until their degradation in the vacuole/lysosome in various organisms, including *A. thaliana* (Kirisako et al., 1999; Kabeya et al., 2000; Yoshimoto et al., 2004; Izumi et al., 2015). Therefore, fluorescently tagged MpATG8 is expected to be a good tool to visualize autophagosome dynamics in *M. polymorpha*. As shown in Figure 2A, monomeric Citrine (mCitrine)-tagged MpATG8a and MpATG8b were localized to punctate structures distributed throughout the cytosol. Fluorescence was also detected in the nucleus (asterisks in Figure 2A), which might reflect a function of the nucleus as a reservoir of MpATG8 as reported in mammalian cells (Huang et al., 2015). As autophagic bodies bearing ATG8 transported to the vacuolar lumen are generally degraded immediately by vacuolar enzymes (Kirisako et al., 1999; Huang et al., 2000), we treated transgenic *M. polymorpha* with concanamycin A (concA), which inhibits acidification of the vacuole and inactivates vacuolar lytic enzymes, to visualize autophagic bodies in the vacuole (Yoshimoto et al., 2004). After treatment with concA, both mCitrine-MpATG8a and mCitrine-MpATG8b were visible as punctate structures in the vacuole (Figure 2C). Vacuolar localization was not observed in mock-treated cells, suggesting that these punctate structures are autophagic bodies. We then investigated whether MpATG8a and MpATG8b are localized to the same structure. We expressed monomeric RFP (mRFP)-tagged MpATG8a and mCitrine-MpATG8b in the same plant and observed strong colocalization at the same punctate structures in the cytosol; colocalization was also observed in the vacuole in concA-treated cells, suggesting that MpATG8a and MpATG8b are localized to the same autophagosomes/autophagic bodies (Figure 2B,D). These observations indicated that MpATG8a and MpATG8b behave in a similar manner to ATG8 in other organisms, and these molecules with fluorescent tags would be useful as autophagosome markers.

### Generation of *atg* Mutants of *M. polymorpha* by Genome Editing

**FIGURE 3:**
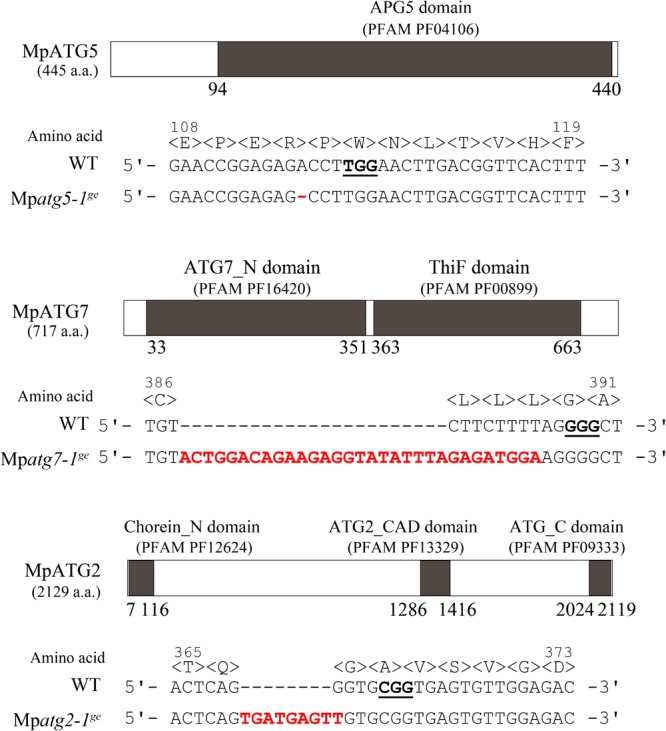
Mutations in Mp*atg5-1^ge^*, Mp*atg7-1^ge^*, and Mp*atg2-1^ge^*. Domain structures of the MpATG5, MpATG7, and MpATG2 proteins and sequences of the mutations in Mp*ATG5*, Mp*ATG7*, and Mp*ATG2* introduced by genome editing. All three mutations result in frame shifts. Underlining indicates protospacer adjacent motif (PAM) sequences. Inserted, deleted, and substituted bases are shown in red.

**FIGURE 4:**
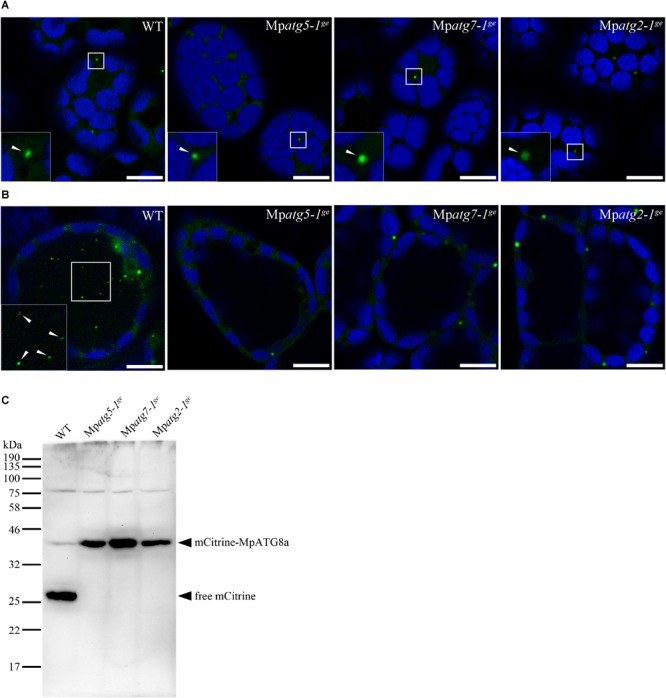
Generation of *atg* mutants of *M. polymorpha*. **(A)** MpATG8a was localized to punctate structures (arrowheads) throughout the cytosol in WT, Mp*atg5-1^ge^*, Mp*atg7-1^ge^*, and Mp*atg2-1^ge^*. Bars = 10 μm. **(B)** mCitrine-MpATG8a was not detected in the vacuole in Mp*atg5-1^ge^*, Mp*atg7-1^ge^*, and Mp*atg2-1^ge^* after concA treatment. Arrowheads indicate punctate structures in the vacuole. The insets are magnified images of the boxed regions. The green and blue pseudocolors indicate fluorescence from mCitrine and autofluorescence of chlorophyll, respectively. Scale bars = 10 μm. **(C)** Five-day-old thalli of WT, Mp*atg5-1^ge^*, Mp*atg7-1^ge^*, and Mp*atg2-1^ge^* expressing mCitrine-MpATG8a were subjected to immunoblotting using the anti-GFP antibody.

To investigate the physiological significance of autophagy in *M. polymorpha*, we generated Mp*atg5*, Mp*atg7*, and Mp*atg2* mutants (hereafter referred to as Mp*atg5-1^ge^*, Mp*atg7-1^ge^*, and Mp*atg2-1^ge^*, respectively) by genome editing using the clustered regularly interspaced short palindromic repeats (CRISPRs)-associated endonuclease Cas9 (CRISPR/Cas9) system (Sugano et al., 2014, 2018). *ATG5, ATG7*, and *ATG2* are required for autophagosome formation, and deletion of these genes causes defects in autophagy in various organisms, including *S. cerevisiae*, mammals, and *A. thaliana* (Tsukada and Ohsumi, 1993; Mizushima et al., 2001; Doelling et al., 2002; Kuma et al., 2004; Komatsu et al., 2005; Thompson et al., 2005; Inoue et al., 2006; Velikkakath et al., 2012). The mutations detected in Mp*atg5-1^ge^*, Mp*atg7-1^ge^*, and Mp*atg2-1^ge^* result in frame shifts, and functional full-length proteins cannot be produced in these mutants (Figure 3). To investigate whether autophagy occurs in these mutants, we observed the dynamics of mCitrine-MpATG8a in these mutants. In wild-type (WT) plants, mCitrine-MpATG8a was localized to the punctate structures in the cytoplasm and vacuolar lumen (Figure 2A,C, 4A). In contrast, vacuolar localization of mCitrine-MpATG8a was not observed in any of the Mp*atg* mutants, while mCitrine-MpATG8a was observed as puncta in the cytoplasm (Figure 4A,B). We then performed a cleavage assay of mCitrine-MpATG8a in the Mp*atg* mutants. As ATG8 is rapidly degraded in the vacuole, whereas mCitrine/YFP is more resistant to lytic enzymes, translocation of mCitrine-MpATG8 to the vacuole can be monitored by examining the accumulation of free mCitrine by immunoblotting (Shintani and Klionsky, 2004; Chung et al., 2010). In WT, two bands were observed via immunoblotting using an anti-green fluorescent protein (GFP) antibody, at approximately 43 and 27 kDa (Figure 4C). The 43 kDa band represented full-length mCitrine-MpATG8a, and the 27 kDa product represented free mCitrine. In contrast, only the 43 kDa product was detected in Mp*atg* mutants, confirming that mCitrine-MpATG8a was not transported into the vacuole in these mutants (Figure 4C). This result indicated that Mp*atg5-1^ge^*, Mp*atg7-1^ge^*, and Mp*atg2-1^ge^* are defective in autophagy and that MpATG8 is transported into the vacuole in an MpATG5-, MpATG7-, and MpATG2-dependent manner in *M. polymorpha.*

**FIGURE 5:**
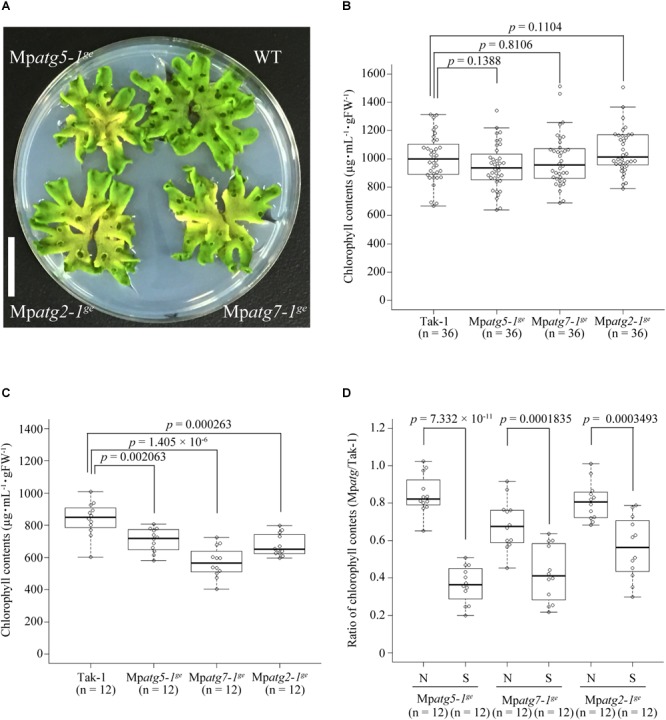
Phenotype of *atg* mutants of *M. polymorpha*. **(A)** 26-day-old thalli grown on 1/2× Gamborg’s B5 medium under continuous light. Scale bar = 2 cm. **(B)** Chlorophyll contents of 5-day-old WT or Mp*atg* mutant thalli before incubation in liquid 1/2× Gamborg’s B5 medium or 10 mM MES. **(C)** Chlorophyll contents of WT and Mp*atg* mutants after incubation in liquid 1/2× Gamborg’s B5 medium for 3 days under continuous light. **(D)** Ratios of chlorophyll contents in Mp*atg* mutants to those in WT, which were incubated in liquid 1/2× Gamborg’s B5 medium under continuous light for 3 days (N, non-starvation) or in 10 mM MES (pH 5.5) for 3 days under dark condition (S, starvation). The boxes and solid lines in the boxes show the first quartile and third quartile, and median values, respectively. The whiskers indicate 1.5× interquartile ranges. *p*-Values were calculated by Welch’s *t-*test. *n*, the number of samples (three thalli were treated as one sample for correct measurement of the fresh weight).

We then observed the macroscopic phenotypes of Mp*atg* mutants. Under normal laboratory conditions, the proximal regions of the thalli of all Mp*atg* mutants exhibited a yellowish chlorotic phenotype, which is similar to the early senescence phenotype of *atg* mutants of *A. thaliana* (Doelling et al., 2002; Hanaoka et al., 2002; Figure 5A). Thus, autophagy appears to also be required for preventing early senescence in *M. polymorpha*. We then investigated whether Mp*atg* mutants are hypersensitive to nutrient starvation, as reported for *atg* mutants of *A. thaliana* (Doelling et al., 2002; Hanaoka et al., 2002). We cultured thalli of *M. polymorpha* in liquid 1/2× Gamborg’s B5 medium under continuous light (control) or in 10 mM 2-(N-morpholino)ethanesulfonic acid (MES) under the dark condition, which induces both bulk autophagy and piecemeal autophagy of chloroplasts in *A. thaliana* (Izumi et al., 2010), and measured chlorophyll contents. Consistent with the early senescence phenotype shown in Figure 5A, Mp*atg* mutants exhibited lower chlorophyll contents after incubation in 1/2× Gamborg’s B5 medium for 3 days under continuous light compared with WT, confirming that autophagy plays critical roles in preventing early senescence in *M. polymorpha* (Figure 5B,C). We also found that Mp*atg* mutants cultured in 10 mM MES for 3 days under dark condition exhibited significantly lower chlorophyll contents than that in WT cultured in 10 mM MES (Figure 5D). These data indicated that autophagy is required for normal response to nutrient starvation in *M. polymorpha*.

## Discussion

*ATG* genes were first identified in *S. cerevisiae*, and most *ATG* genes have now been shown to be conserved in various lineages of eukaryotes. In this study, we showed that charophyte species and *M. polymorpha* also possess a set of genes for the core autophagy machinery with lower redundancy than those of *P. patens* and *A. thaliana*. This result suggests that the core autophagy machinery has been expanded during land plant evolution. Some of the *ATG* genes, such as *ATG8* and *ATG18*, are reported to have multiplied in various organisms, including land plants. Intriguingly, *M. polymorpha* also harbors multiple *ATG8* and *ATG18* genes, and the plant-unique ATG18 group comprising the BCAS3 domain is conserved in *M. polymorpha*, some charophytes, and the chlorophyte *C. subellipsoidea*. The distribution of the plant-unique ATG18 suggests that this type of ATG18 was acquired before the emergence of Streptophyta, and secondary losses of this gene occurred independently during plant evolution, although its molecular function remains unknown. Moreover, it remains unclear whether the members of ATG8 and ATG18 are functionally differentiated in plants. In *A. thaliana*, knockdown of *ATG18a*, one of eight *ATG18* homologs, results in similar but weaker phenotypes to those of mutants of other *ATG* genes, suggesting that ATG18a plays a major role in autophagy, although the other seven *ATG18* homologs could have a partly redundant function (Xiong et al., 2005; Kang et al., 2018). In addition to its localization to the PAS, Atg18 of *S. cerevisiae* is also localized to the vacuolar membrane via phosphatidylinositol 3,5-bisphosphate binding and acts in retrograde transport from the vacuole, which is independent of autophagy (Dove et al., 2004; Efe et al., 2007). *ATG18* homologs in *A. thaliana* might also have autophagy-independent functions. In mammals, two groups of ATG8-related proteins, the LC3 and GABARAP/GATE-16 subfamilies, are both localized to the autophagosomes but play distinct roles in autophagosome formation (Kabeya et al., 2004; Weidberg et al., 2010). The LC3 group is involved in the elongation of the phagophore membrane, whereas GABARAP/GATE-16 plays an essential role in a later stage of autophagosome maturation (Weidberg et al., 2010). *Caenorhabditis elegans* possesses two *ATG8* homologs: *LGG-1* and *LGG-2*. LGG-1 is involved in the formation of autophagosomes. In contrast, LGG-2 mediates the maturation of autophagosomes and facilitates their tethering with lysosomes through interaction with VPS39 (Manil-Segalen et al., 2014). In plants, distinct binding affinities of potato ATG8 members with PexRD54, an effector protein in the pathogenic fungus *Phytophthora infestans*, have been reported (Dagdas et al., 2016). Thus, the plant ATG8 and ATG18 groups could comprise functionally differentiated members; this possibility should be verified in future studies.

Although most of the core machinery of autophagy is conserved in plants, *ATG10* homologs were not detected in two charophyte species. Loss of *ATG10* has been observed in various lineages of eukaryotes, some of which utilize a noncovalent complex of ATG5 and ATG12 without the C-terminal glycine residue (Pang et al., 2019). Given that ATG10 mediates covalent linkage between ATG12 and ATG5 at the C-terminal glycine residue of ATG12, secondary loss of ATG10 could be associated with conversion from covalent to noncovalent ATG12–ATG5 complexes during evolution, which could be followed by deletion of the glycine residue of the C-terminus of ATG12. Consistent with this notion, we did not detect *ATG10* homologs in two charophyte species, one of which (*C. braunii*) possesses ATG12 without C-terminal glycine (Tables 1, 2). Further characterization of ATG12 and ATG5 in *C. braunii* would be needed to verify the possible convergence of the ubiquitin-like conjugation system involving ATG12 and ATG5 across a wide range of eukaryotic lineages.

*M. polymorpha* has been recognized as a new model for analyzing the developmental processes of land plants (Bowman, 2016; Bowman et al., 2017; Ishizaki, 2017). This plant would also be useful for analyses of molecular mechanisms of autophagy due to its low genetic redundancy and widely available molecular genetic tools (Ishizaki et al., 2016; Bowman et al., 2017). In the interest of understanding the molecular mechanisms of autophagy in plants, it would be beneficial to reveal the molecular functions of ATG8 members, given that nonplant ATG8 acts at various steps of autophagy, such as expansion and closure of the isolation membrane, cargo recognition, and transport of autophagosomes to the vacuole/lysosome (Abeliovich et al., 2000; Nakatogawa et al., 2007; Fujita et al., 2008a; Kimura et al., 2008; Xie et al., 2008; Noda et al., 2010; Manil-Segalen et al., 2014; Nguyen et al., 2016; Tsuboyama et al., 2016). Although several ATG8-interacting proteins, such as ATI1/2, ATI3, DSK2, NBR1, ORM1/2, PUX, RPN10, and TSPO, which are involved in autophagy induced by certain stresses, have been identified (Svenning et al., 2011; Vanhee et al., 2011; Honig et al., 2012; Zhou et al., 2013, 2014, 2018; Hachez et al., 2014; Michaeli et al., 2014; Marshall et al., 2015, 2019; Hafren et al., 2017; Nolan et al., 2017; Yang et al., 2019), it remains unclear how various substrates are selectively targeted by autophagy in plants (Yoshimoto and Ohsumi, 2018). Given that *M. polymorpha* possesses fewer copies of *ATG8* than other model plants, this plant would be useful for revealing the molecular functions of ATG8 members and functional diversification of ATG8 in land plants.

In this study, we succeeded in visualizing autophagosomes using fluorescently tagged ATG8 proteins in *M. polymorpha*. Both MpATG8a and MpATG8b were localized to punctate structures in the cytosol and vacuole. Deletion of Mp*ATG2*, Mp*ATG5*, or Mp*ATG7* resulted in defective transport of MpATG8a into the vacuole, indicating that vacuolar transport of ATG8 is autophagy-dependent in *M. polymorpha*, as reported in other organisms. Punctate localization of MpATG8a was also detected even in the Mp*atg* mutants, and similar localization is also observed in Arabidopsis *atg* mutants (Yoshimoto et al., 2004; Kang et al., 2018). ATG8/LC3 is reported to be incorporated into protein aggregates in an autophagy-independent manner in mammalian cells (Kuma et al., 2007; Tanida et al., 2008). Therefore, it is highly likely that MpATG8a also aggregates in the cytosol independently of autophagic activities in the Mp*atg* mutants. It would be also possible that some population of fluorescently tagged MpATG8a/b-positive puncta observed in WT plants represents unfunctional protein aggregates, which should be verified in future studies.

The autophagy-defective mutants of *M. polymorpha* exhibited an early senescence-like phenotype and hypersensitivity to nutrient starvation (Figure 5), which resembles the phenotypes observed in *atg* mutants of other plants (Doelling et al., 2002; Hanaoka et al., 2002; Mukae et al., 2015; Wada et al., 2015). In *A. thaliana*, salicylic acid (SA) signaling is involved in early senescence in *atg* mutants (Yoshimoto et al., 2009). Although the relevance of SA signaling to the senescence of Mp*atg* mutants remains unknown, autophagy might play a common role in preventing senescence among land plants.

A forward genetic approach using *M. polymorpha* would also be effective to reveal the molecular mechanisms of autophagy in land plants, as in other systems (Tsukada and Ohsumi, 1993; Thumm et al., 1994; Harding, 1995; Tian et al., 2010; Morita et al., 2018). Its haploid-dominant life cycle and low genetic redundancy could make this plant even more amenable to forward genetic analyses than other model plants, including *A. thaliana* (Ishizaki et al., 2016). Screening of mutants defective in autophagy and functional analyses of obtained factors would facilitate understanding of the molecular mechanisms of autophagy in land plants. In conclusion, *M. polymorpha* is a suitable system for analyzing autophagy in land plants. Further studies in this plant will contribute to revealing the molecular mechanisms of autophagy in plants, which would also be useful to gain insights into how the autophagy machinery has been functionally diversified and how autophagy has been recruited to support plant physiology.

## Materials and Methods

### Identification of Orthologs for Core Autophagy Machinery

Amino acid sequences of core autophagy machinery in *K. nitens, P. patens*, and *M. polymorpha* were obtained in MarpolBase^1^ using *ATG* genes of *A. thaliana* (Li et al., 2014; Shemi et al., 2015; Liu et al., 2018) as queries. *ATG* genes in *C. braunii* were searched in the *C. braunii* portal site^2^. For core autophagy machinery orthologs in *C. reinhardtii* except for *ATG11*, we referred to the previous study (Jiang et al., 2012; Shemi et al., 2015). The *ATG11* homolog in *C. reinhardtii* was searched in MarpolBase. For *ATG17, ATG29*, and *ATG31*, whose homologs have not been identified in *A. thaliana* thus far, *ATG* genes in *S. cerevisiae* were used as queries. *ATG10* and *ATG12* homologs in *S. pratensis, N. mirabilis, C. orbicularis*, and *M. viride* were searched in the transcriptome database in MarpolBase. *ATG18* homologs in *D. salina, V. carteri, C. subellipsoidea* C-169, *M. pusilla* CCMP1545, and *O. lucimarinus* were searched in Phytozome v12.1.6^3^. *ATG18* homologs in *O. tauri* were searched in MarpolBase. A domain search was performed using SMART^4^ (Letunic et al., 2014; Letunic and Bork, 2017). The accession numbers and amino acid sequences analyzed in this study are included in the Supplementary Material. We followed the nomenclature proposed in Bowman et al. (2016) for nomenclature of genes, proteins, and mutants of *M. polymorpha*.

### Phylogenetic Analysis of ATG18

Amino acid sequences of ATG18 in various plant species were aligned with ClustalX 2.1 (Larkin et al., 2007), and alignment gaps were removed using Gblocks^5^. Phylogenetic analysis was performed using PhyML 3.0^6^ (Guindon et al., 2010) under the LG+G+I+F model, which was selected by Smart Model Selection in PhyML (Lefort et al., 2017). Bootstrap analysis was performed by resampling 1,000 sets. The sequences used in the phylogenetic analysis and the alignment from which gaps were removed were included in the Supplementary Material.

### Vector Construction

Genomic sequences of Mp*ATG8a* (Mapoly0001s0494.1) and Mp*ATG8b* (Mapoly0027s0034.1) were amplified by PCR from genomic DNA prepared from gemmae of *M. polymorpha* accession Takaragaike-1 (Tak-1, male) (Ishizaki et al., 2008), and the amplified products were subcloned into pENTR/D-TOPO (Invitrogen) according to the manufacturer’s instructions. To construct mCitrine- and mRFP-MpATG8, genomic sequences comprising the protein-coding regions and 3′-flanking sequences (2 kb) were amplified with the *Sma*I site followed by a flexible linker sequence (Gly–Gly–Ser–Gly) attached at the 5′-end and subcloned into the pENTR vector. Then, cDNA for mRFP or mCitrine containing the *Sma*I site at the 5′-end was inserted into the *Sma*I site of pENTR vectors containing the Mp*ATG8* genes using the In-Fusion HD Cloning System (Clontech) according to the manufacturer’s instructions. The 5 kb 5′-sequence [promoter + 5′-untranslated region (UTR)] of each MpATG8 was then amplified and inserted into the *Sma*I site of the mRFP/mCitrine-MpATG8 vectors. The resultant chimeric genes were then introduced into pMpGWB301 (mCitrine-tagged MpATG8a and MpATG8b) or pMpGWB101 (mRFP-MpATG8a) (Ishizaki et al., 2015) using the Gateway LR Clonase^TM^ II Enzyme Mix (Invitrogen) according to the manufacturer’s instructions. To construct CRISPR/Cas9 vectors, two complementary oligonucleotides in the sequences of Mp*ATG2*, Mp*ATG5*, and Mp*ATG7* were synthesized and annealed, and the resulting double-stranded fragments were subcloned at the *Bsa*I site of the pMpGE_En03 vector (Sugano et al., 2018). The resultant gRNA cassette flanked by the *att*L1 and *att*L2 sequences in pMpGE_En03 were then introduced into the pMpGE010 vector (Sugano et al., 2018) using the Gateway LR Clonase II Enzyme Mix. The list of primer sequences used in this study is included in the Supplementary Material.

### Plant Material and Transformation

The *M. polymorpha* accession Tak-1 was grown asexually and maintained on 1/2× Gamborg’s B5 medium containing 1.4% agar at 22°C under continuous white light. Transformation was performed as previously described (Kubota et al., 2013). Transformants were selected on plates containing 10 mg/l hygromycin B and 250 mg/l cefotaxime for the pMpGWB101 and pMpGE010 vectors and 0.5 μM chlorsulfuron and 250 mg/l cefotaxime for the pMpGWB301 vector.

### Confocal Laser Scanning Microscopy

Five-day-old thalli grown on 1/2× Gamborg’s B5 medium containing 1.4% agar at 22°C under continuous white light were used for observation. Dorsal thallus tissues were observed using an LSM 780 confocal microscope (Carl Zeiss) as previously described (Kanazawa et al., 2016). For concA treatment, 4-day-old thalli were incubated in liquid 1/2× Gamborg’s B5 medium plus 1 μM concA (Santa Cruz Biotechnology, sc-202111) for 14 h at 22°C under continuous white light. concA was dissolved in dimethyl sulfoxide (DMSO) at 1 mM as a stock solution. For the mock treatment, samples were treated with DMSO at a concentration equal to that used for the inhibitor-treated samples.

### Identification of Mutation Points

For genotyping of mutants generated by CRISPR/Cas9, total RNA was extracted from 5-day-old thalli of Mp*atg5-1^ge^*, Mp*atg7-1^ge^*, and Mp*atg2-1^ge^* using the RNeasy Plant Mini Kit (Qiagen) and used as a template for reverse transcription using SuperScript III Reverse Transcriptase (Invitrogen) and the oligo (dT) (18-mer) primer according to the manufacturer’s instructions. Mutations in the obtained cDNA fragments were analyzed by direct sequencing.

### Immunoblot Analysis

Five-day-old thalli were used for the immunoblot analysis. One hundred milligrams of plants were homogenized in 200 μl of grinding buffer [50 mM HEPES–KOH, pH 7.5, 340 mM sorbitol, 5 mM MgCl_2_, and 1× Complete^TM^ Protease Inhibitor Cocktail (Roche)] for each genotype and centrifuged at 1,000 × *g* for 10 min. The supernatants were centrifuged at 3,000 × *g* for 10 min, and the resulting supernatants were used for immunoblotting. The polyclonal anti-GFP antibody (Kanazawa et al., 2016) was purified by affinity column chromatography using the GST-mCitrine protein bound to the HiTrap^TM^ NHS-activated HP Column (GE Healthcare) and used at 500× dilution. The peroxidase-conjugated donkey anti-rabbit immunoglobulin antibody (GE Healthcare) was used as the secondary antibody. Signals were detected using Immobilon Western Chemiluminescent HRP Substrate (Merck).

### Measurement of Chlorophyll Content

Five-day-old thalli were incubated in 1 ml of liquid 1/2× Gamborg’s B5 medium for 3 days under continuous light or in 10 mM MES (pH 5.5) for 3 days under dark condition, and chlorophyll was extracted by soaking in 500 μl *N,N*-dimethylformamide overnight. Calculation of chlorophyll concentrations was done according to Porra et al. (1989). Three thalli were treated as one sample for correct measurement of fresh weights.

## Data Availability

The raw data supporting the conclusions of this manuscript will be made available by the authors, without undue reservation, to any qualified researcher.

## Author Contributions

TN and TU designed the research and wrote the manuscript. TN performed a major part of the experiment and analyzed the data. TK and NM prepared the anti-GFP antibody. TK assisted with vector construction. HT and TU supervised the study.

## Conflict of Interest Statement

The authors declare that the research was conducted in the absence of any commercial or financial relationships that could be construed as a potential conflict of interest.

## Footnotes

1 http://marchantia.info/

2 https://bioinformatics.psb.ugent.be/orcae/overview/Chbra

3 https://phytozome.jgi.doe.gov/pz/portal.html

4 http://smart.embl-heidelberg.de/

5 http://molevol.cmima.csic.es/castresana/Gblocks_server.html

6 http://www.atgc-montpellier.fr/phyml/

## Supplementary Material

DATA SET S1 This data set contains all amino acid sequences analyzed in this study, the alignment generated for phylogenetic analysis, and lists of primers and accession numbers.
